# Quantitative phosphoproteomic analysis reveals chemoresistance-related proteins and signaling pathways induced by rhIL-6 in human osteosarcoma cells

**DOI:** 10.1186/s12935-021-02286-z

**Published:** 2021-10-30

**Authors:** Rui Zhang, Huan Wang, Erliang Li, Yonghong Wu, Yanhua Wen, Chenyu Li, Bo Liao, Qiong Ma

**Affiliations:** Orthopedic Oncology Institute, Department of Orthopedic Surgery, Tangdu Hospital, Fourth Military Medical University, Xi’an, China

**Keywords:** Chemoresistance, Lobaplatin, rhIL-6 intervention, Osteosarcoma, Proteomics, Phosphorylation, p-filamin-C, MAPK signaling

## Abstract

**Background:**

IL-6 plays a pivotal role in resistance to chemotherapeutics, including lobaplatin. However, the underlying mechanisms are still unclear. This study was to investigate the changes in phosphoproteins and their related signaling pathways in the process of IL-6-induced chemoresistance to lobaplain in osteosarcoma cells.

**Methods:**

We performed a quantitative phosphoproteomic analysis of the response of SaOS-2 osteosarcoma cells to recombinant human IL-6 (rhIL-6) intervention prior to lobaplatin treatment. The cells were divided into the control group (Con), the lobaplatin group (Lob), and the rhIL-6-and-lobaplatin group (IL-6). Three biological replicates of each group were included. The differentially expressed phosphoproteins were subjected to Gene Ontology (GO) and Kyoto Encyclopedia of Genes and Genomes (KEGG) enrichment analyses. Netphos 3.1 was used for the prediction of kinases, and STRING was used for the visualization of protein–protein interactions. The conserved motifs surrounding the phosphorylated residues were analyzed using the motif-x algorithm. Western blot analysis was performed to verify the differential expression of p-FLNC, its predicted kinase and the related signaling pathway. The results of the bioinformatic analysis were validated by immunohistochemical staining of clinical specimens.

**Results:**

In total, 3373 proteins and 12,183 peptides, including 3232 phosphorylated proteins and 11,358 phosphorylated peptides, were identified and quantified. Twenty-three significantly differentially expressed phosphoproteins were identified in the comparison between the IL-6 and Lob groups, and p-FLNC ranked second among these phosphoproteins. GO and KEGG analyses revealed the pivotal role of mitogen-activated protein kinase signaling in drug resistance induced by rhIL-6. Four motifs, namely, -SPxxK-, -RxxSP-, -SP-, and -SPK-, demonstrated higher expression in the IL-6 group than in the Lob group. The western blot analysis results verified the higher expression of p-FLNC, AKT1, and p-ERK and the lower expression of p-JNK in the IL-6 group than in the Con and Lob groups. The immunohistochemical staining results showed that p-FLNC, AKT1 and p-ERK1/2 were highly expressed in platinum-resistant clinical specimens but weakly expressed in platinum-sensitive specimens, and platinum-resistant osteosarcoma specimens demonstrated weak expression of p-JNK.

**Conclusions:**

This phosphoproteomic study is the first to reveal the signature associated with rhIL-6 intervention before lobaplatin treatment in human osteosarcoma cells. p-FLNC, AKT1, and MAPK signaling contributes to resistance to lobaplatin in osteosarcoma SaOS-2 cells and may represent molecular targets to overcome osteosarcoma chemoresistance.

**Supplementary Information:**

The online version contains supplementary material available at 10.1186/s12935-021-02286-z.

## Background

Osteosarcoma is the most common malignant bone tumor in adolescents and is considered insensitive to radiotherapy and immunotherapy. The clinical treatment for osteosarcoma includes aggressive and complete surgical resection and accompanying preoperative and postoperative chemotherapy [1, 2]. Platinum-based drugs are commonly used chemotherapeutic agents for osteosarcoma and have been developed from the first-generation chemotherapeutic agent cisplatin to the new agent lobaplatin, which exhibits better antitumor activity and low toxicity [3]. However, many patients still develop resistance to lobaplatin after a period of treatment.

IL-6 is a crucial cytokine involved in the pathogenesis of many chronic inflammatory diseases, including cancer. This cytokine can activate STAT3 through the classical pathway and trans-signaling pathway, resulting in either anti-inflammatory or proinflammatory effects [4]. Targeting IL-6 could be an effective strategy for overcoming stroma-induced chemotherapeutic resistance to tocilizumab in gastric cancer [5]. Additionally, according to previous studies, IL-6 is involved in resistance to chemotherapy in osteosarcoma cells in vitro [6–8]. However, to date, specific changes in phosphoproteins at the post-translational level have not been discovered.

Proteins should first be modified after translation to perform their specific functions, and site-specific phosphorylation is the most pivotal post-translational modification that can regulate the function of proteins by altering their activities, stability or interactions. Therefore, dysregulation of site-specific phospho-signaling is a hallmark of many cancers [9]. Phosphoproteomic analyses offer great advantages in the investigation of the differences in both phosphoproteins and their related signaling pathways. In this study, we explored the differential expression of phosphoproteins during rhIL-6 intervention before treatment with the chemotherapeutic agent lobaplatin in SaOS-2 osteosarcoma cells. The potential site-specific phosphoproteins and the related signaling pathways that may contribute to lobaplatin resistance in osteosarcoma cells were revealed.

## Materials and methods

### Osteosarcoma cell culture and treatment

The osteosarcoma cell line SaOS-2 was kindly provided by Procell Life Science & Technology Co., Ltd. and cultured in Dulbecco’s modified Eagle’s medium (Gibco, USA) supplemented with 10% fetal bovine serum (Gibco) and 1% penicillin–streptomycin (HyClone, USA). The cells were grown in a humidified atmosphere with 5% carbon dioxide at 37 °C. Cells grown to 80% confluence were used in the subsequent experiments. Trypsin–EDTA solution (0.25%, HyClone, USA) was used for cell detachment when needed. The rhIL-6 cytokine (PeproTech, USA) was diluted in PBS containing 5% trehalose. Lobaplatin (MCE, USA, Cat# 135558-11-1) was diluted in ddH_2_O. The cells were divided into the following three groups: the Con group (osteosarcoma cells treated with the solvent of both rhIL-6 and lobaplatin), the Lob group (osteosarcoma cells treated first with a solvent of rhIL-6 and then with lobaplatin at a concentration of 10 μg/mL for 24 h) and the IL-6 group (osteosarcoma cells pretreated with 60 ng/mL rhIL-6 eight hours before the lobaplatin treatment).

The anti-FLNC monoclonal antibody (Cat# ab180941), anti-AKT1 monoclonal antibody (Cat# ab108202), anti-P38 monoclonal antibody (Cat# ab170099), anti-p-P38 monoclonal antibody (Cat# ab178867), and anti-GAPDH monoclonal antibody (Cat# ab8245) were purchased from Abcam; the anti-JNK antibody (Cat# 9252T), anti-p-JNK antibody (Cat# 4668T), anti-ERK1/2 antibody (Cat# 4695T), and anti-p-ERK1/2 antibody (Cat# 4370T) were purchased from Cell Signaling Technology; the anti-p-FLNC antibody (Cat# bs-13183R) was purchased from Bioss Antibodies; and the HRP-conjugated secondary antibodies (Cat# 31430, 31460) were purchased from Thermo Fisher Scientific.

### Phosphoproteomic analysis

#### Sample preparation

Osteosarcoma cells (9 × 10^6^) were seeded in 15-cm dishes: in total, 18 culture dishes were used to establish three replicates per sample. After the previously described treatment, the cells were washed three times with ice-cold PBS and collected by scraping into several centrifuge tubes. The cells were centrifuged at 4 °C and 1000 rpm three times for 5 min each, and the PBS buffer was removed. Then, the cells were lysed in precooled RIPA lysis and extraction buffer (Thermo Scientific, USA, Cat# 89900) supplemented with 1% protease inhibitor cocktail (Cell Signaling, Cat# 5871) and 1% phosphatase inhibitor (Cell Signaling, Cat# 5872). The lysates were centrifuged at 14,000*g* for 10 min at 4 °C. The supernatants were transferred to new tubes and quantified using a BCA Protein Assay Kit (Piece, USA). The protein lysates were stored at − 20 °C. Twenty micrograms of protein from each sample was mixed with 6 × loading buffer and boiled for 5 min. The proteins were separated by 10% SDS-PAGE and stained with Coomassie Blue R-250.

In total, 600 μg of protein from each sample was added to 30 μL of SDT buffer containing 100 mM dithiothreitol (DTT), 4% SDS, and 150 mM Tris–HCl. Uric acid (UA) buffer was used to remove DTT and other low-molecular-weight components by repeated ultrafiltration. Then, 100 mM iodoacetamide was added, and the samples were incubated in darkness for 30 min. The filters were washed several times with UA buffer and TEAB buffer. The protein suspensions were digested with trypsin (Promega, USA) overnight at 37 °C, and the filtrates were collected. The peptide content was estimated by measuring the optical density of a 0.1% (g/L) solution under 280 nm UV light irradiation using an extinction coefficient of 1.1 calculated, based on the frequencies of tryptophan and tyrosine in vertebrate proteins.

#### Enrichment of phosphorylated peptides

A 100 μg peptide mixture of each protein sample was labeled using TMT reagents (Thermo Fisher Scientific, USA). The peptides in the Con group were labeled with TMT-126, TMT-127N, and TMT-127C; the peptides in the Lob group were labeled with TMT-128N, TMT-128C, and TMT-129N; and the peptides in the IL-6 group were labeled with TMT-129C, TMT-130N and TMT-130C. Each group included three biological replicates. The labeled peptides were combined and desalted using a C18 cartridge. The peptide mixtures were processed with a HiSelect TiO_2_ Phosphopeptide Enrichment Kit (Thermo Fisher Scientific, A32993). The TiO_2_ flowthrough and wash fractions were pooled, and the phosphopeptides were enriched with a HiSelect Fe-NTA Phosphopeptide Enrichment Kit (Thermo Fisher Scientific, A32992). The TiO_2_ eluent and Fe-NTA eluent were dried via vacuum centrifugation at 45 °C and then dissolved in 0.1% formic acid buffer.

### LC–MS analysis

Each eluent was injected twice for nanoliquid chromatography–tandem mass spectrometry (nanoLC-MS/MS) analysis. The peptide mixtures were loaded onto a C18 reversed-phase analytical column (Thermo Fisher Scientific, USA, P/N164943) in buffer A (0.1% formic acid) and separated with a linear gradient of buffer B (80% acetonitrile and 0.1% formic acid) at a regular flow rate of 300 nL/min. The linear gradient was as follows: 6% buffer B for 5 min, 6–28% buffer B for 215 min, 28–38% buffer B for 10 min, 38–99% buffer B for 5 min, and 99% buffer B for 10 min. A Q Exactive Plus mass spectrometer (Thermo Fisher Scientific, USA) was coupled to an Easy-nLC chromatography system (Thermo Fisher Scientific, USA) and operated in the positive ion mode. The MS data were acquired using a data-dependent method that could dynamically choose the most abundant precursor ions from full scans (ranging from 350 to 1800 *m*/*z*) for higher-energy collisional dissociation (HCD) fragmentation. The survey scans were performed at a resolution of 70,000 at 200 *m*/*z* with an automatic gain control (AGC) target of 3e6 and a maximum injection time (IT) of 50 ms. The MS2 scans were performed at a resolution of 35,000 for HCD spectra at 200 *m*/*z* with an AGC target of 2e5 and a maximum IT of 120 ms with an isolation width of 2 *m*/*z*. Only ions with a charge state between 2 and 6 and a minimum intensity of 2e3 were selected for fragmentation. The duration of the dynamic exclusion of the selected ions was 30 s, and the normalized collision energy was set to 30 eV.

The raw MS/MS data files were processed using the MASCOT engine (Matrix Science, London, UK; version 2.6, RRID: SCR_014322) embedded in Proteome Discoverer 2.1 and searched against the UniProt_HomoSapiens_20386_20180905 database. A maximum of 2 missed cleavages was permitted. A precursor mass tolerance of 10 ppm was specified, and a tolerance of 0.05 Da was set for the MS2 fragments. In addition to the TMT labels, carbamidomethyl (C) was set as a fixed modification. The variable modifications included oxidation, acetylation, and phosphorylation. A peptide and protein false discovery rate of 1% was enforced using a reverse database search strategy. Phosphopeptides with a fold change > 1.2, maximum number of missed cleavages ≤ 2, and p-value < 0.05 were considered differentially expressed.

### Clustering, GO and KEGG analyses

A bioinformatic analysis was performed to compare the IL-6 group and the Lob group. The quantitative data of the target protein set were normalized. Matplotlib software was used to classify the samples and protein expression in two dimensions simultaneously (distance algorithm: Euclidean, connection method: average linkage), and a hierarchical clustering heatmap was generated for visualization. The GO annotation of the target protein sets was performed with Blast2GO [10]. Specifically, the target protein sets were compared with the appropriate protein sequence database using the sequence comparison tool NCBI BLAST + (ncbi-blast − 2.3.0 +) on the Linux server, and the first 10 aligned sequences with san E-value ≤ 1e−3 were retained for the subsequent analysis. The GO entries associated with the target protein set and the highest bit score alignment sequence were extracted using the Blast2GO Command Line (download link: www.geneontology.org). During the annotation, the similarity between the target protein sequence and the aligned sequence, the reliability of the source of the GO entry, and the structure of the GO-directed acyclic graph were comprehensively considered, and the exact GO entry extracted in the mapping process was assigned to the target protein sequence. After the annotation was completed, to further improve the annotation efficiency, the EBI database was searched for conserved motifs matching the target protein using InterProScan [11], and the motif-related functional information was annotated to the target protein sequence. ANNEX was used to further supplement the annotation information, and then links between different GO categories were established to improve the accuracy of the annotations.

Orthologous genes and their products with similar functions in the same pathway were grouped and assigned the same KO (or K) label. During the KEGG pathway annotation, the target protein sequences were classified according to their KO labels based on the KEGG gene database, and the information of the pathway in which the sequence participated was obtained according to the KO classification using KEGG Orthology and Links Annotation (KOALA) software [12].

### Motif analysis

A motif analysis of the differentially expressed phosphorylated peptides between the IL-6 group and the Lob group was performed using the motif-x algorithm. The prealigned sequences were 13-amino-acid peptides with serine, threonine, or tyrosine arranged in the center as the key residue. The human background was selected, and the score threshold was set to 1.0e-6. Then, the logos of different motifs were visualized using Momo software (version 5.0.1) [13].

### Kinase prediction and PPI network analysis

Kinase prediction was performed using the web resource NetPhos 3.1 (http://www.cbs.dtu.dk/services/NetPhos/), which predicts the serine, threonine, or tyrosine phosphorylation sites in eukaryotic proteins using ensembles of neural networks after inputting the substrate sequences in FASTA format. The residue to be predicted was set to either serine or threonine according to the MS results. For each residue, only the best prediction was chosen. Kinases with a score higher than 0.5 were selected.

The PPIs were retrieved from the STRING database (http://string-db.org/), which shows the experimental data and predicted data by computational algorithms [14]. The protein IDs were input, and *Homo sapiens* was selected as the species. Protein IDs with a score higher than 0.9 were selected. The degrees of connectivity of each predicted kinase of the differentially expressed phosphoproteins between the IL-6 group and the Lob group were calculated to verify the importance of the kinases in the PPI network. The kinase with connections to the highest number of other predicted kinases was considered the most important kinase.

### Western blot analysis

Cell cultures were harvested in RIPA buffer containing protease and phosphatase inhibitors (Cell Signaling, USA; Cat# 5871, 5872). After quantification with a BCA assay kit (Piece, USA), 60 μg of protein was separated on 6–10% SDS-PAGE gels and then transferred to PVDF membranes (Millipore, USA) for the phosphoproteins. The membranes were blocked with 5% nonfat milk powder or BSA and incubated with primary antibodies (anti-FLNC–1:10,000, anti-p-FLNC–1:500, anti-AKT1–1:5000, anti-ERK1/2–1:1000, anti-p-ERK1/2–1:2000, anti-JNK–1:1000, anti-p-JNK–1:1000, and anti-GAPDH–1:7500) at 4 °C overnight. The antibody-bound proteins were incubated with an HRP-conjugated secondary antibody at room temperature for 1 h and then detected using ECL reagents (Pierce, USA) on a chemiluminescence imaging system (BIORAD, USA) as previously described [15].

### Clinical specimens

The studies involving patient specimens were approved by the Ethics Committee of Tangdu Hospital, and written informed consent was provided by the patients or their relatives. Clinical osteosarcoma specimens were collected from forty osteosarcoma patients who were treated with platinum-based chemotherapy and then underwent resection of visible tumors in the Orthopedic Department of Tangdu Hospital between January 2010 and May 2016. Patients with tumors exhibiting greater than 50% regression were considered chemotherapy-sensitive [16]. In addition, five-year follow-up data were considered; twenty-four specimens were classified into the chemotherapy-resistant group, and the other sixteen specimens were classified into the chemotherapy-sensitive group. The clinicopathological data of the osteosarcoma patients were recorded.

### Immunohistochemical staining

Paraffin-embedded tissue specimens were sliced into 4-μm sections (LEICA, Germany) and heated at 60 °C for two hours. Then, the sliced specimens were dewaxed in xylene and rehydrated in graded ethanol solutions following standard procedures. Citrate buffer (10 mM) was used for antigen retrieval, and 3% H_2_O_2_ was used to block endogenous peroxidase activity. The slides were incubated with primary antibodies (anti-FLNC–1:250, anti-p-FLNC–1:400, anti-AKT1–1:500, anti-ERK1/2–1:250, anti-p-ERK1/2–1:200, anti-JNK–1:100, anti-p-JNK–1:50, anti-P38–1:100, and anti-p-P38–1:500) at 4 °C overnight. After incubation with the secondary antibody (GK500710, Gene Tech, China) at room temperature for 30 min, the slides were treated with DAB substrate. The staining images were acquired under an Olympus BX51 microscope.

### Statistical analysis

Each experiment was performed at least three times. The statistical analysis of the differences between two groups was conducted with Student’s *t*-tests. One-way ANOVA with Tukey’s post hoc test was performed for comparisons among multiple groups. For the analysis of the immunohistochemical staining, the mean optical density (MOD) was calculated using a Mann–Whitney U test. The data were analyzed with GraphPad Prism (version 7, RRID: SCR_002798). P-values < 0.05 were considered indicative of significant differences.

## Results

### Proteomic screening of phosphorylation in osteosarcoma cells

**Fig. 1:**
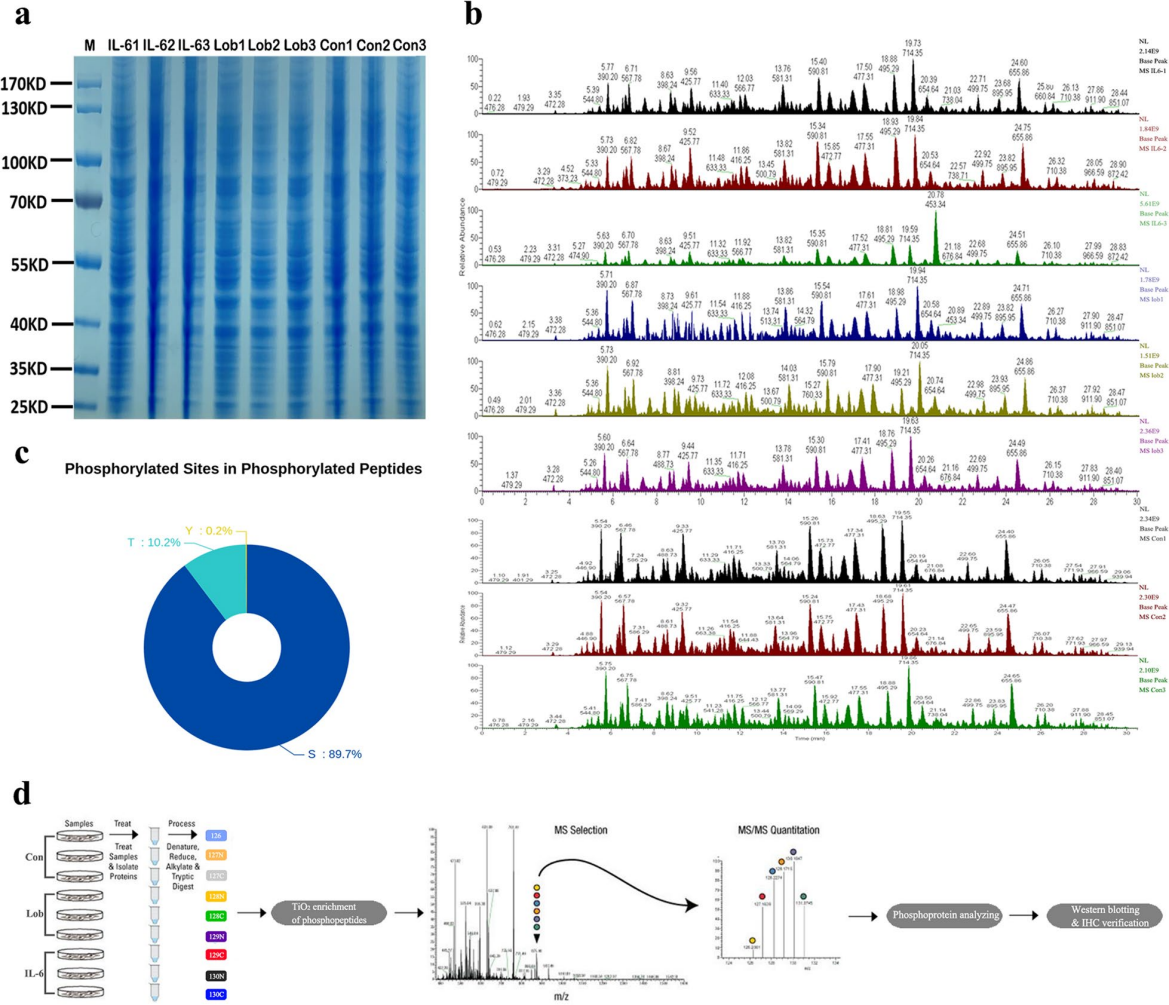
LC–MS/MS-based quantitative phosphoproteomic profiling of phosphorylation in SaOS-2 osteosarcoma cells. **a** Protein expression profiling of osteosarcoma cells in the Con group, Lob group, and IL-6 group. Twenty micrograms of protein was loaded, and the gel was stained with Coomassie Blue R-250 after electrophoresis. **b** Base peak chromatograms demonstrating the correspondence of peptides in each group. **c** Phosphorylation patterns of the amino acids serine, threonine, and tyrosine. **d** Experimental scheme of the quantitative phosphoproteomic analysis of osteosarcoma cells with IL-6 intervention before lobaplatin treatment

The total protein expression levels in each group of SaOS-2 osteosarcoma cells were determined by sodium dodecyl sulfate–polyacrylamide gel electrophoresis (SDS-PAGE) and Coomassie Brilliant Blue staining to eliminate the effects of differential protein expression on the comparison of phosphoprotein abundances; the protein expression levels derived from the whole cell lysates of the Con group, the Lob group, and the IL-6 group were similar (Fig. 1a). In addition, base peak chromatograms were generated to assess the correspondence of peptides between different samples, and the findings showed good quality both within and between groups (Fig. 1b). The phosphorylation levels of proteins in each group were then evaluated. The percentages of phosphorylation on the amino acids threonine, serine, and tyrosine are shown in Fig. 1c, indicating the highest modification level on serine (89.7%) and the lowest modification level on tyrosine (0.2%).

**Fig. 2:**
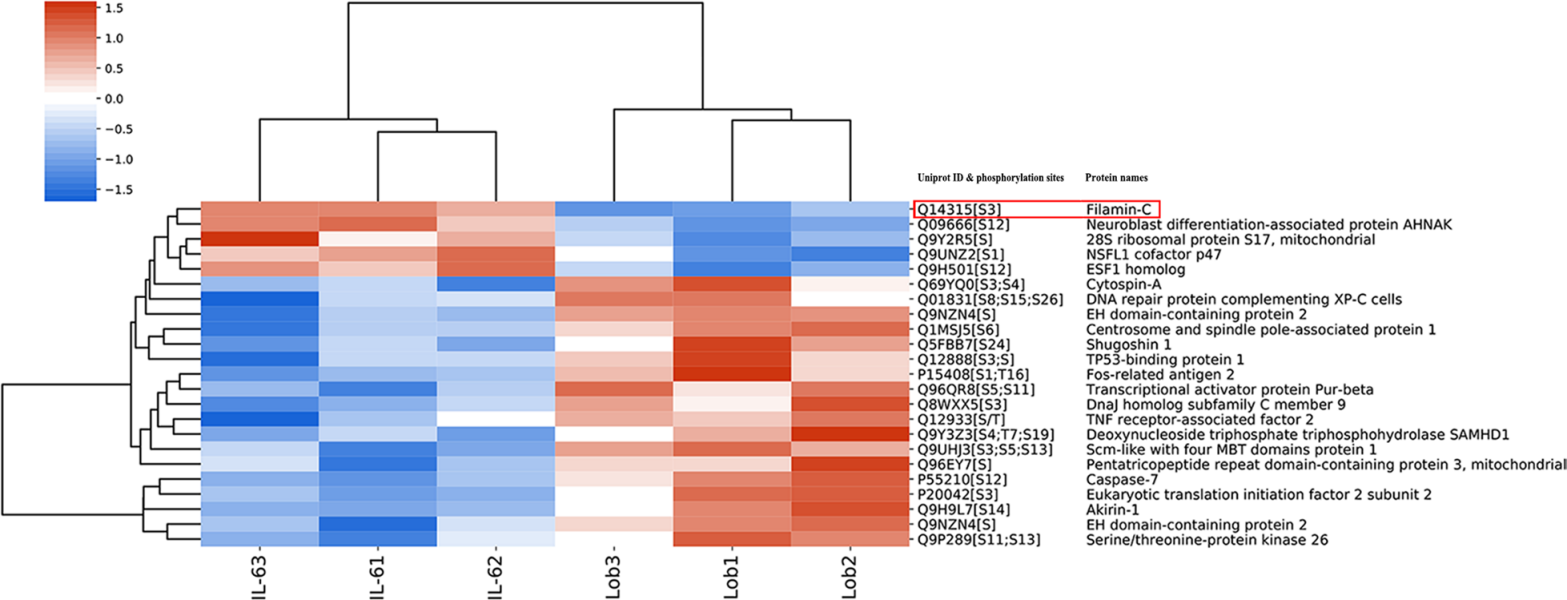
Hierarchical clustering of differentially expressed phosphoproteins and their specific phosphorylation sites between the Lob group and the IL-6 group. Each group contained three replicates. A heatmap was generated to visualize the distribution of phosphoproteins in the different groups. Red, blue and white indicate increased expression, decreased expression, and a lack of recorded information in the protein extracts, respectively

A schematic outline of the phosphoproteomic analysis of chemoresistant osteosarcoma cells treated with rhIL-6 and lobaplatin is depicted in a flow chart (Fig. 1d). In total, 3373 proteins and 12,183 peptides were identified, including 3232 phosphorylated proteins and 11,358 phosphorylated peptides. Twenty-three significantly differentially expressed phosphorylated peptides (5 upregulated by more than 1.2-fold and 18 downregulated by less than 0.83-fold, with 39 phosphorylation sites (p < 0.05)) between the IL-6 group and the Lob group were identified. A hierarchical clustering heatmap was generated to show the phosphoproteins and their specific phosphorylation sites and, thus, provide a better visualization of the overall phosphoproteomic changes. p-FLNC ranked as the second-most highly expressed phosphoprotein (Fig. 2, Table 1; Additional file 1).

**Table 1:** Differentially expressed phosphoproteins between the IL-6 group and the Lob group

Phosphoprotein names	Protein accessions	Gene Names	Fold-change	Up/down	P-value
28S ribosomal protein S17, mitochondrial	Q9Y2R5	MRPS17	1.610	Up	0.0284
Filamin-C	Q14315	FLNC	1.264	Up	0.000664
NSFL1 cofactor p47	Q9UNZ2	NSFL1C	1.248	Up	0.0337
ESF1 homolog	Q9H501	ESF1	1.233	Up	0.00661
Neuroblast differentiation-associated protein AHNAK	Q09666	AHNAK	1.214	Up	0.00374
Caspase-7	P55210	CASP7	0.828	Down	0.00626
Cytospin-A	Q69YQ0	SPECC1L	0.825	Down	0.0233
Scm-like with four MBT domains protein 1	Q9UHJ3	SFMBT1	0.819	Down	0.00401
Eukaryotic translation initiation factor 2 subunit 2	P20042	EIF2S2	0.805	Down	0.0170
EH domain-containing protein 2	Q9NZN4	EHD2	0.804	Down	0.0182
Centrosome and spindle pole-associated protein 1	Q1MSJ5	CSPP1	0.798	Down	0.0132
DnaJ homolog subfamily C member 9	Q8WXX5	DNAJC9	0.793	Down	0.0184
Deoxynucleoside triphosphate triphosphohydrolase SAMHD1	Q9Y3Z3	SAMHD1	0.787	Down	0.0343
Akirin-1	Q9H9L7	AKIRIN1	0.785	Down	0.0156
TP53-binding protein 1	Q12888	TP53BP1	0.781	Down	0.0337
Serine/threonine-protein kinase 26	Q9P289	STK26	0.778	Down	0.0331
Transcriptional activator protein Pur-beta	Q96QR8	PURB	0.768	Down	0.0114
DNA repair protein complementing XP-C cells	Q01831	XPC	0.762	Down	0.0448
Pentatricopeptide repeat domain-containing protein 3, mitochondrial	Q96EY7	PTCD3	0.744	Down	0.0371
Shugoshin 1	Q5FBB7	SGO1	0.738	Down	0.0259
Fos-related antigen 2	P15408	FOSL2	0.719	Down	0.0138
TNF receptor-associated factor 2	Q12933	TRAF2	0.634	Down	0.0385

### Functional annotation of the differentially expressed phosphoproteins (DEPs)

**Fig. 3:**
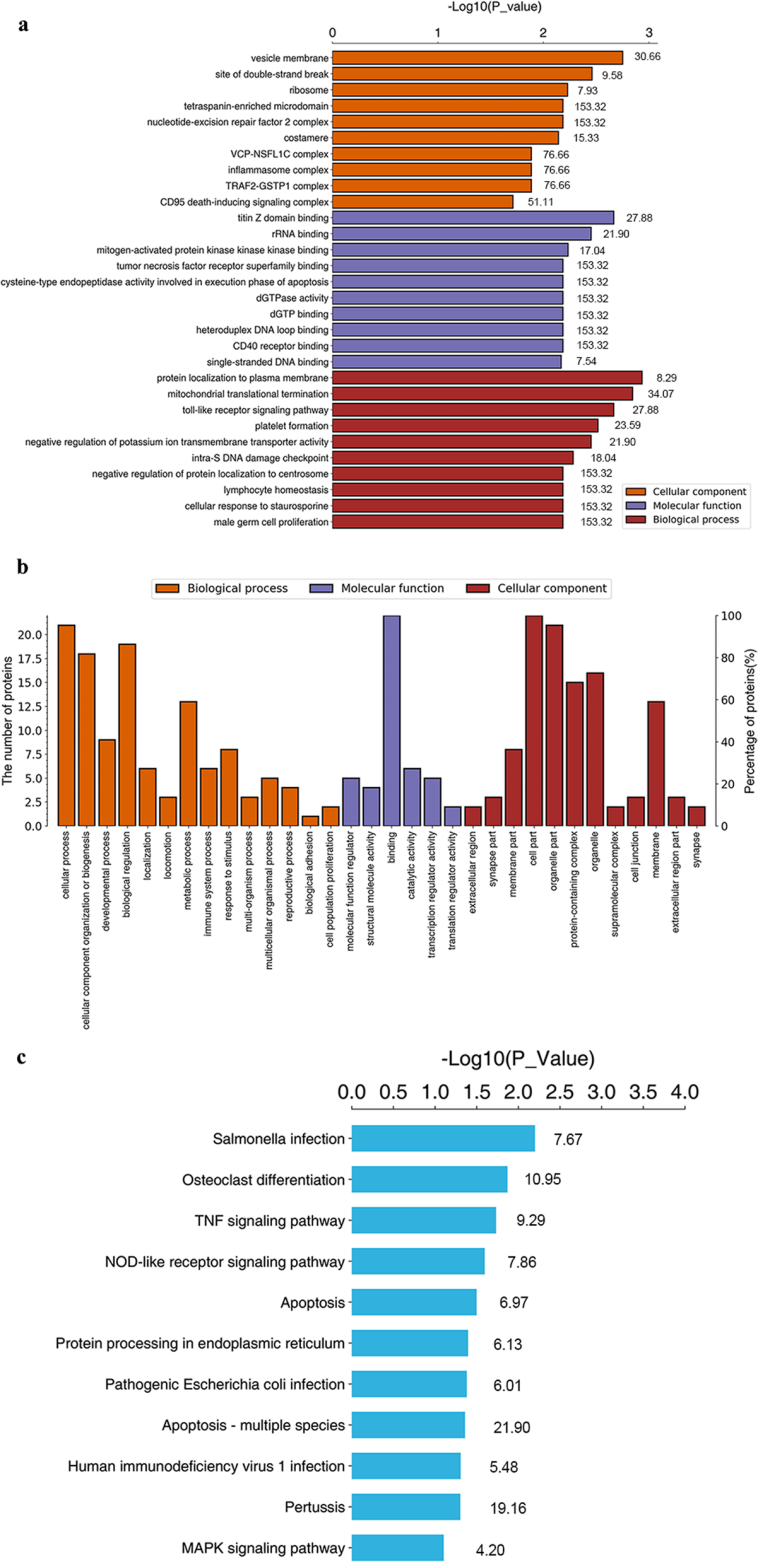
GO and KEGG pathway analyses of differentially expressed phosphoproteins between the Lob group and the IL-6 group. Three biological replicates were performed for each group. **a** GO analysis of phosphoproteins differentially regulated in the IL-6 group compared with those in the Lob group. Fisher’s exact test was used. BP: biological process, MF: molecular function, CC: cellular component. The numbers adjacent to each bar are enrichment factors representing the significance and reliability of the phosphoproteins enriched in the term. **b** Overall level 2 GO enrichment analysis in the biological process, molecular function, and cellular component categories. The left vertical axis shows the numbers of differentially expressed phosphoproteins annotated to a specific term. The right axis shows the percentage of phosphoproteins in the specific item relative to the total number of differentially expressed phosphoproteins. **c** KEGG pathway analysis showing the signaling pathways up- or downregulated between the IL-6 group and the Lob group. The number adjacent to each bar is the enrichment factor representing the significance of each pathway

To investigate the differences in the differentially expressed phosphoproteins between the Lob group and the IL-6 group, GO and KEGG enrichment analyses and related analyses were performed. Among the significantly upregulated phosphoproteins, the most enriched GO term in the biological process category was protein localization to the plasma membrane (GO: 0072659, enrichment factor = 8.29, p = 0.00116), and in the molecular function category, the most enriched GO term was kinase binding (GO: 0019900, enrichment factor = 5.58, p = 0.0489). Among the significantly downregulated phosphoproteins, the main enriched GO terms were protein localization to the plasma membrane (GO: 0072659, enrichment factor = 8.29, p = 0.00116), toll-like receptor (GO: 0002224, enrichment factor = 27.88, p = 0.00216) and Hippo signaling (GO: 0035329, enrichment factor = 8.76, p = 0.0212) in the biological process category; mitogen-activated protein kinase binding (GO: 0031435, enrichment factor = 17.04, p = 0.00583) and dGTPase activity (GO: 0008832, enrichment factor = 153.32, p = 0.00652) in the molecular function category; and inflammasome complex (GO: 0061702, enrichment factor = 76.66, p = 0.0130), CD95 death-inducing signaling complex (GO: 0,031,265, enrichment factor = 51.11, p = 0.0194), and gap junction (GO: 0005921, enrichment factor = 38.33, p = 0.0258) in the cellular compartment category (Fig. 3a, b; Table 2).

**Table 2:** Enrichment of phosphoproteins and signaling pathways between the IL-6 group and Lob group

Terms	Protein Number	P-value	FDR	Enrich-ment Factor	Phosphoprotein names
GO-UP					
BP					
Protein localization to plasma membrane	4	0.00116	0.150	8.29	p-filamin-C; p-EH domain-containing protein 2; p-cytospin-A; p-serine/threonine-protein kinase 26
MF					
Kinase binding	2	0.0490	0.392	5.58	p-filamin-C; p-shugoshin 1
GO-DOWN					
MF					
Toll-like receptor	2	0.00216	0.349	27.88	p-caspase-7; p-TNF receptor-associated factor 2
Hippo signaling	2	0.0212	0.171	8.76	p-caspase-7; p-serine/threonine-protein kinase 26
Mitogen-activated protein kinase binding	2	0.00583	0.430	17.04	p-serine/threonine-protein kinase 26; p-TNF receptor-associated factor 2
CC					
Inflammasome complex	1	0.0130	0.116	76.66	p-caspase-7
CD95 death-inducing signaling complex	1	0.0194	0.171	51.11	p-caspase-7
Gap junction	1	0.0258	0.210	38.33	p-cytospin-A
KEGG pathways					
TNF signaling pathway	2	0.0190	0.186	9.29	p-caspase-7; p-TNF receptor-associated factor 2
NOD-like receptor signaling pathway	2	0.0260	0.394	7.86	p-TP53-binding protein 1; p-TNF receptor-associated factor 2
Apoptosis	2	0.0326	0.186	6.97	p-caspase-7; p-TNF receptor-associated factor 2
MAPK signaling pathway	2	0.0808	0.289	4.20	p-filamin-C; p-TNF receptor-associated factor 2

*BP* biological processes, *MF* molecular functions, *CC* cellular components, *FDR* false discovery rate

GO and KEGG pathway enrichment results were analyzed with Fisher’s exact test, and p < 0.05 was considered significant

The KEGG pathway analysis revealed that the pathway annotations TNF signaling pathway, NOD-like receptor signaling pathway, apoptosis and MAPK signaling pathway were enriched (including two proteins in each pathway) between the Lob group and IL-6 group, with enrichment factors of 9.29, 7.86, 6.97, and 4.20, respectively (Fig. 3c; Table 2).

### Phosphorylation motifs in lobaplatin-resistant osteosarcoma cells

**Fig. 4:**
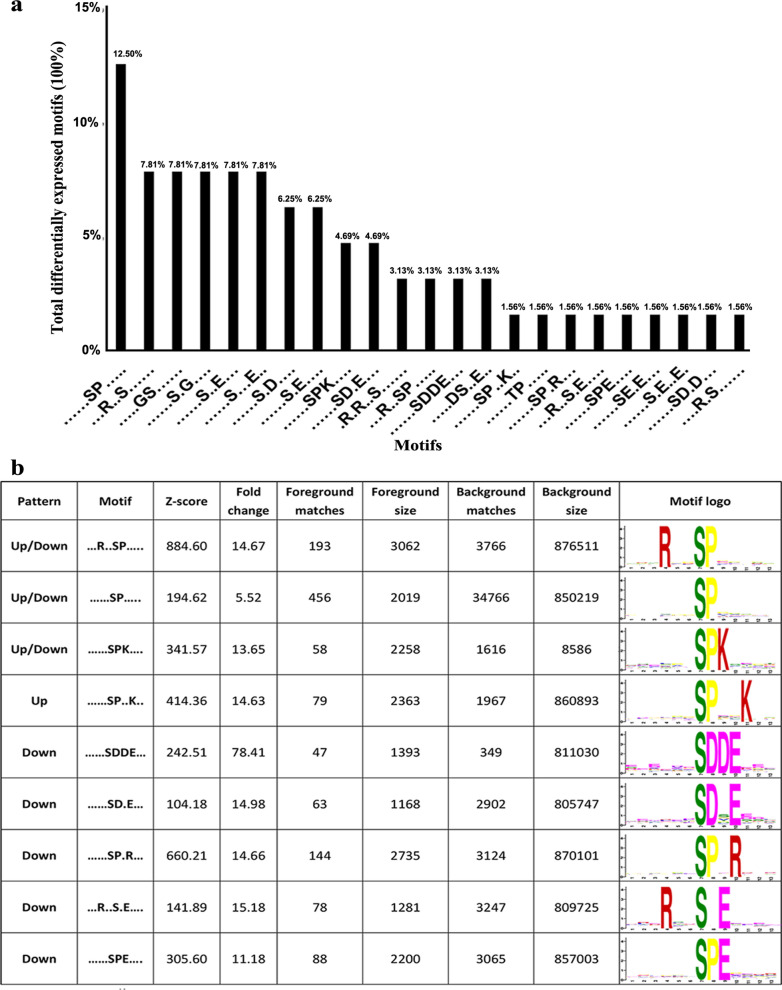
Differentially expressed motifs between the IL-6 group and the Lob group. **a** Twenty-three conserved motifs (specifically, 22 p-Ser motifs and 1 p-Thr motif) were identified and enriched among the phosphosites. The vertical bars represent the percentage of the specific motif relative to the total number of identified differentially expressed motifs between the two groups. **b** High-scoring motifs differentially expressed between the IL-6 group and the Lob group. Four motifs (-RxxSP-, -SP-, SPK-, and -SPxxK-) were upregulated in the IL-6 group; five motifs (SDDE-, -SDxE, -SPxR-, -RxxSxE-, and -SPE-) were downregulated. Three motifs (-RxxSP-, -SP-, and SPK-) were identified as enriched in both the upregulated and downregulated phosphosites

The motifs surrounding phosphorylation sites are generally believed to be specific substrates for kinases. To characterize the motifs identified in the Lob group and IL-6 group, the differentially expressed phosphorylated peptides were analyzed using the motif-x algorithm. Twenty-three motifs (specifically, twenty-two phosphorylated (p)-Ser motifs and one p-Thr motif) were identified among the differentially expressed phosphosites between the Lob group and the IL-6 group (Fig. 4a). Among the twenty-three differentially expressed motifs, nine p-Ser motifs were high-scoring motifs and are listed in Fig. 4b. Among these 9 motifs, the motif -SPxxK- was enriched in the upregulated phosphoproteins, five motifs (-SDDE-, SDxE-, -SPxR-, -RxxSxE-, and -SPE-) were enriched in the downregulated phosphoproteins in the IL-6 group compared with the Lob group, and the motifs -RxxSP-, -SP-, and -SPK- were enriched in both the upregulated and downregulated phosphosites. The motif logos were generated using the sequence logo tool from MEME (http://alternate.meme-suite.org/tools/meme).

### Kinase prediction and PPI analysis

**Fig. 5:**
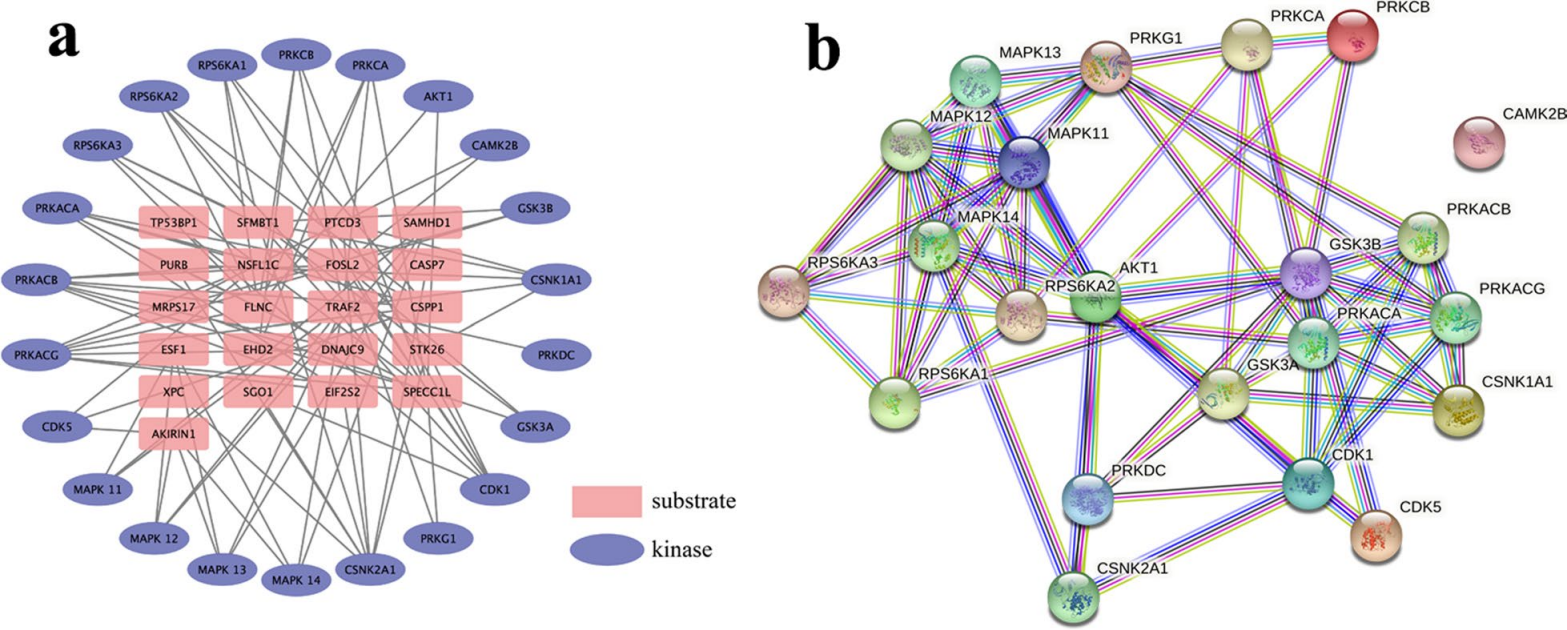
Prediction and PPI network of the upstream kinases of the differentially expressed phosphoproteins between the IL-6 group and the Lob group. **a** In total, 93 kinase-substrate interactions were predicted using NetPhos 3.1 according to the differentially expressed phosphoproteins and the specific phosphosites. **b** PPIs of the predicted kinases. PPI network analysis was performed using the STRING database. The blue lines show the interactions obtained from the curated database; the purple lines show the interactions that were experimentally determined; the dark green lines indicate the gene neighborhood; the light green lines show the text mining interactions; the red lines indicate gene fusions; and the light violet lines show protein homologies

The kinases of the differentially expressed phosphoproteins between the IL-6 group and the Lob group were predicted using NetPhos 3.1 while comprehensively considering the phosphorylation sites identified by a tandem mass tag (TMT)-based quantitative phosphoproteomic analysis and the prediction scores. In total, twenty-two kinases were predicted to regulate the differentially expressed phosphosites, and the relationships between the predicted kinases and the significantly differentially expressed phosphoproteins were visualized with Cytoscape (Fig. 5a). The protein–protein interaction (PPI) networks of these predicted kinases were visualized using STRING (http://string-db.org/cgi/input.pl). The most enriched highly connected kinases between the IL-6 group and the Lob group were as follows: rac-alpha serine/threonine protein kinase (UniProtKB-P31749), which interacted with eleven predicted kinases; glycogen synthase kinase-3 beta (UniProtKB-P49841), which interacted with ten predicted kinases; and mitogen-activated protein kinase 14 (UniProtKB-Q16539), which interacted with nine predicted kinases (Fig. 5b; Table 3). Site-specific kinase and substrate predictions and network analysis demonstrated extensive interactions among the predicted kinases.

**Table 3:** Highly connected predicted kinases in the protein–protein interaction in the STRING database

Node kinase	UniProt ID	Protein name	Gene name
RAC-alpha serine/threonine-protein kinase (eleven connected)	O15264	Mitogen-activated protein kinase 13	MAPK13
	Q15759	Mitogen-activated protein kinase 11	MAPK11
	P53778	Mitogen-activated protein kinase 12	MAPK12
	Q14157	Mitogen-activated protein kinase 14	MAPK14
	P78527	DNA-dependent protein kinase catalytic subunit	PRKDC
	P68400	Casein kinase II subunit alpha	CSNK2A1
	P49840	Glycogen synthase kinase-3 alpha	GSK3A
	P49841	Glycogen synthase kinase-3 beta	GSK3B
	P06493	Cyclin-dependent kinase 1	CDK1
	Q00535	Cyclin-dependent-like kinase 5	CDK5
	P05771	Protein kinase C beta type	PRKCB
Glycogen synthase kinase-3 beta (ten connected)	P05771	Protein kinase C beta type	PRKCB
	P17252	Protein kinase C alpha type	PRKCA
	P31749	RAC-alpha serine/threonine-protein kinase Anillin	AKT1
	Q15418	Ribosomal protein S6 kinase alpha-1	RPS6KA1
	O43663	DNA-dependent protein kinase catalytic subunit	PRKDC
	P49840	Glycogen synthase kinase-3 alpha	GSK3A
	Q00535	Cyclin-dependent-like kinase 5	CDK5
	P22694	cAMP-dependent protein kinase catalytic subunit beta	PRKACB
	P22612	cAMP-dependent protein kinase catalytic subunit gamma	PRKACG
	P48729	Casein kinase I isoform alpha	CSNK1A1
Mitogen-activated protein kinase 14 (nine connected)	Q15759	Mitogen-activated protein kinase 11	MAPK11
	P53778	Mitogen-activated protein kinase 12	MAPK12
	O15264	Mitogen-activated protein kinase 13	MAPK13
	Q15418	Ribosomal protein S6 kinase alpha-1	RPS6KA1
	Q15349	Ribosomal protein S6 kinase alpha-2	RPS6KA2
	P51812	Ribosomal protein S6 kinase alpha-3	RPS6KA3
	P31749	RAC-alpha serine/threonine-protein kinase	AKT1
	P68400	Casein kinase II subunit alpha	CSNK2A1
	Q13976	cGMP-dependent protein kinase 1	PRKG1

### Verification of the TMT-based phosphoproteomic analysis results by western blot analysis

**Fig. 6:**
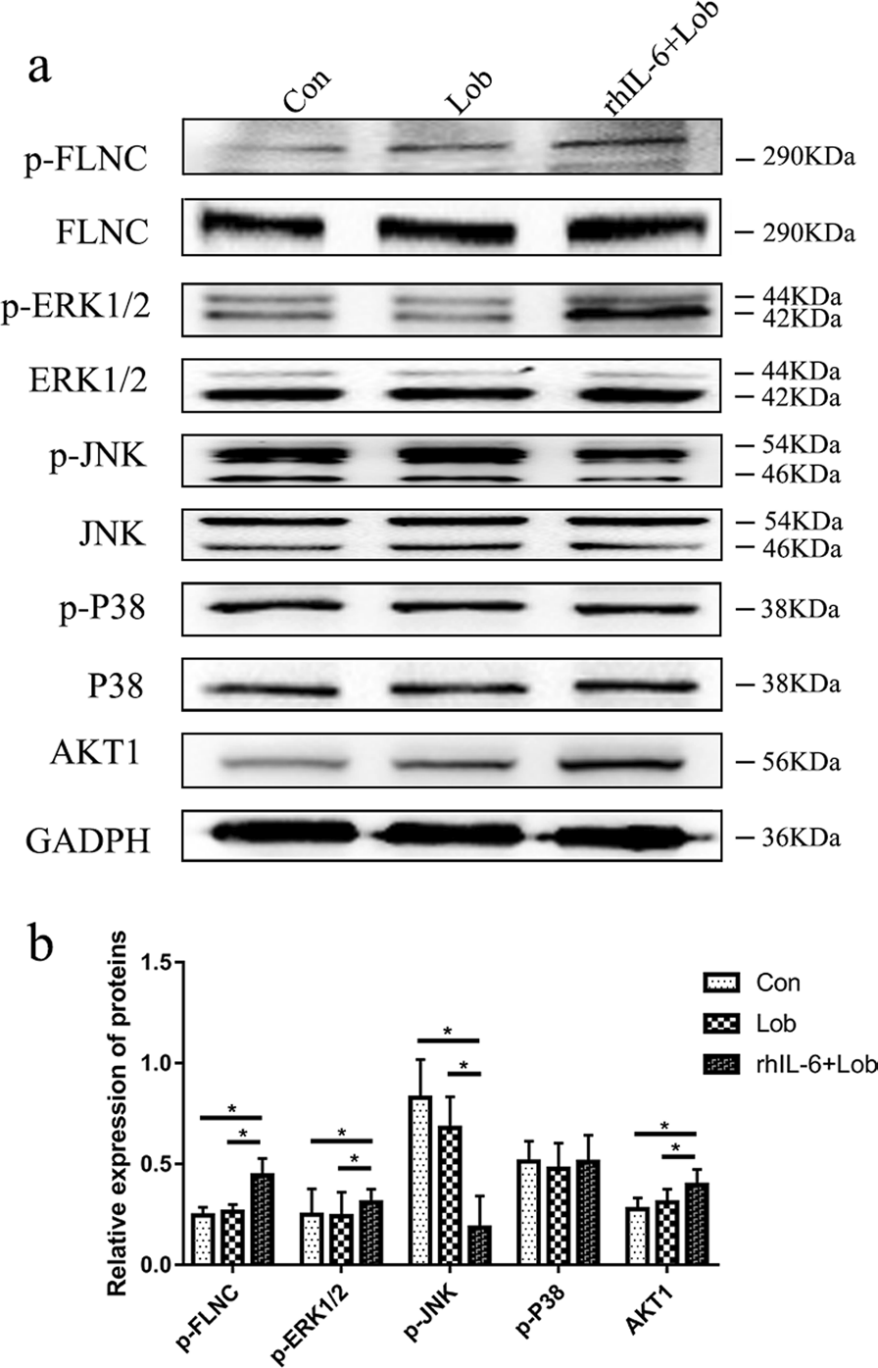
Expression levels of p-FLNC and its predicted kinases AKT1, p-ERK1/2, p-JNK and p-P38 in SaOS-2 osteosarcoma cells. **a** Western blot analysis bands showing higher expression of p-FLNC, AKT1, and p-ERK1/2 in the IL-6 group and lower expression of p-JNK and similar expression of p-P38 between the IL-6 group and the Con and Lob groups. Cells were divided into the Con, Lob, and IL6 groups. **b** ImageJ was used to calculate the density of the bands. The values of each phosphoprotein were normalized against their total proteins, while the value of AKT1 was normalized against GAPDH. Significant differences are indicated. *p < 0.05

The phosphoprotein p-filamin-C was differentially upregulated between the IL-6 group and the Lob group, and its predicted kinase rac-alpha serine/threonine protein kinase was analyzed. Western blot analysis showed that the osteosarcoma cells in the IL-6 group exhibited high levels of p-filamin-C, AKT1 and p-extracellular signal-regulated kinase 1/2 (p-ERK1/2), indicating that during rhIL-6 treatment, which results in lobaplatin resistance, elevated AKT1 promotes a high level of p-filamin-C and activates the MAPK signaling pathway (Fig. 6). This finding also verified the KEGG pathway analysis results, showing that filamin-C was enriched in the MAPK signaling pathway (Table 2).

### Immunohistochemical (IHC) staining of clinical specimens

**Fig. 7:**
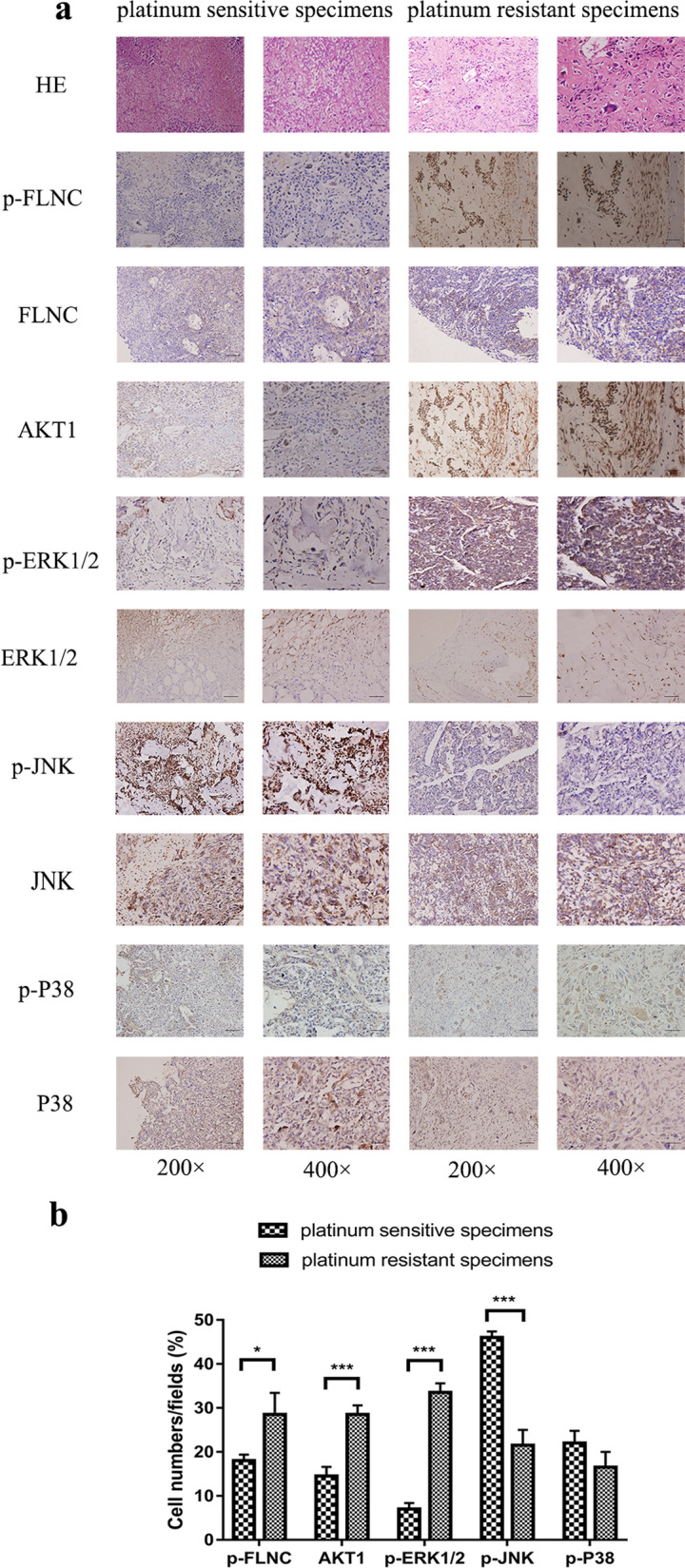
Expression levels of p-FLNC and its predicted kinases AKT1, p-ERK1/2, p-JNK and p-P38 in platinum-sensitive and platinum-resistant osteosarcoma specimens. **a** Representative HE and immunohistochemical staining of platinum-sensitive and platinum-resistant specimens. **b** Quantification of the above phosphorylated proteins and AKT1 using ImageJ. Scale bars = 50 μm (magnification, 400 ×) and 100 μm (magnification, 200 ×). **p < 0.01, ***p < 0.001

In total, forty osteosarcoma specimens (from sixteen patients with resistance to platinum-based chemotherapy and twenty-four chemotherapy-sensitive patients) were obtained to further confirm the quantitative phosphoproteomic analysis results. The patients’ clinical data are recorded in Additional files 2 and 3. Hematoxylin and eosin (HE) staining showed increased necrosis in the chemotherapy-sensitive specimens compared with the chemotherapy-resistant specimens. Immunohistochemical staining of p-filamin-C and its predicted kinase AKT1, p-ERK1/2, p-JNK and p-P38 was performed. Regarding p-filamin-C, twelve chemotherapy-resistant osteosarcoma specimens exhibited strong positive staining, three exhibited moderate positive staining, and one exhibited negative staining. Regarding AKT1 in the same specimens, ten exhibited strong positive staining, four exhibited moderate positive staining and two exhibited negative staining. Regarding p-ERK1/2, thirteen exhibited strong positive staining, and three exhibited moderate staining. Additionally, regarding p-JNK, stronger positive staining was observed in the chemotherapy-sensitive specimens, and no significant difference was detected in p-P38 between the sensitive and resistant specimens (Fig. 7; Additional file 4).

## Discussion

Previous studies revealed the pivotal role of the cytokine IL-6 in the process of chemotherapeutic resistance [17–19]. However, no systematic mechanistic investigation has been conducted from a phosphoproteomic perspective. Phosphorylation functions as a switch for protein activity and is the main mechanism of signal transmission. In addition, phosphorylation is a crucial step in the regulation of pathophysiological processes in various cancers, and numerous drugs and drug candidates target protein kinases [20]. Phosphoproteomic analyses can enrich our knowledge of phosphorylated proteins and identify and quantify phosphorylation sites, thereby providing insight into the specific mechanisms of biological processes, such as proliferation, metastasis, and drug resistance [21]. In this study, we explored the differentially expressed site-specific phosphoproteins, their potential kinases, and the related signaling pathways during rhIL-6 intervention before lobaplatin treatment.

This study is the first to use a TMT-based quantitative phosphoproteomics approach to examine the role of post-translational changes in IL-6-induced lobaplatin resistance in osteosarcoma cells. We identified 3,232 phosphorylated proteins and 11,358 phosphorylated peptides. Regarding the IL6-induced changes in phosphoproteins, we compared the IL-6 group with the Lob group and identified twenty-three significantly differentially expressed phosphoproteins that were relatively tightly related to the effects of IL-6 on lobaplatin resistance.

The GO enrichment analysis suggested that IL-6 pretreatment before lobaplatin treatment significantly regulated kinase binding, Toll-like receptors, Hippo signaling, and mitogen-activated protein kinase binding (all p-values < 0.05) in osteosarcoma cells. Toll-like receptors (TLRs) are membrane-bound pattern recognition receptors that can recruit proinflammatory cytokines and costimulatory molecules through NFκB and MAP kinase signaling [22]. Moreover, TLRs mediate immune evasion, which can result in autophagy dysregulation and chemoresistance [23]. Hippo signaling regulates diverse biological processes, and its dysfunction is related to many cancers [24]. Considerable evidence has demonstrated that the deregulation of YAP/TAZ signaling may play a key role in intrinsic and acquired resistance to various chemotherapies [25]. Regarding the enrichment in MAPK binding, emerging evidence suggests that this event can modulate drug resistance and sensitivity in various cancers [26].

The KEGG pathway analysis provided further insight. The pathway annotations NOD-like receptor (NLR) signaling pathway, TNF signaling pathway, apoptosis and MAPK signaling pathway were enriched. NLRs are considered regulators of tumorigenesis, cancer cell stemness and chemoresistance. The inappropriate activation of NLRs modulates the tissue microenvironment and potentiates the risk for cancers [27, 28]. TNF signaling leads to cell survival, proliferation and differentiation. However, excessive or aberrant activation of TNF-α signaling can lead to chronic inflammation and other pathological complications [29]. The MAPK signaling pathway is a highly conserved signaling pathway involving JNK, ERK1/2, p38, and ERK5 that mediates metabolic reprogramming, cell survival, and cell differentiation. Constitutive activation of this pathway plays a role in cancer development and progression, including autophagy dysregulation and therapeutic resistance [30–32]. The MAPK signaling pathway also participates in crosstalk with the NLR and TNF signaling pathways identified above as enriched [33, 34]. Phosphoproteins, such as p-CASP7, p-FLNC, p-TRAF2, and p-TP53BP1, which are involved in the abovementioned enriched pathways, may be targets for lobaplatin resistance.

Among the identified phosphoproteins, p-filamin-C exhibited the second-highest level in the osteosarcoma cells treated with rhIL-6 prior to lobaplatin compared with those treated with lobaplatin alone. Filamin C is a dimeric actin-binding protein that can regulate remodeling of the actin cytoskeleton and act as a scaffold for signaling proteins, including tyrosine kinases, phosphatases, and GTPases. Therefore, filamins are believed to connect adhesive receptors to signal transduction and the cytoskeleton [35]. High filamin-C expression has been reported to enhance the invasiveness of glioblastoma and predict poor outcomes [36]. Moreover, filamin-C can interact with and activate MEK1/2 and ERK1/2 to promote the progression of hepatocellular carcinoma [37]. In contrast, another study showed that the downregulation of filamin-C by acetylated Siah2 increased the invasiveness of gastric cancer cells [38]. Collectively, the above results suggest that filamin-C acts as a double-edged sword with both detrimental and beneficial roles in patients with cancer and that its role may depend on the type of cancer. However, knowledge regarding the role of phosphorylated filamin-C in cancers is limited. In our study, the p-filamin-C level in rhIL-6- and lobaplatin-treated osteosarcoma cells, which are resistant to lobaplatin as described in a previous study, was higher than that in cells treated with lobaplatin alone. In addition, its predicted kinase PKB (also called AKT1) showed the highest connectivity with other predicted kinases of the differentially expressed phosphorylated proteins between the Lob group and the IL-6 group. Moreover, p-filamin-C was enriched in the MAPK signaling pathway (ko04010), as shown by KEGG pathway analysis. MAPKs can phosphorylate substrates with the motif -SP- [39]. Differential expression of this motif between the Lob group and the IL-6 group was observed, indicating that MAPKs might be activated by IL-6-induced chemoresistance to lobaplatin. Therefore, p-filamin-C is expected to be a candidate target for lobaplatin resistance in osteosarcoma. Western blot analysis and immunohistochemical staining were performed to verify the results of the phosphoproteomic and subsequent bioinformatic analyses, and the levels of p-filamin-C, AKT1 and p-ERK1/2 in the rhIL-6-pretreated osteosarcoma cells were found to be higher than those in the cells treated with lobaplatin alone, while lower expression of p-JNK and no significant change in the level of p-P38 were observed. In the clinical specimens from osteosarcoma patients, most chemotherapy-resistant specimens exhibited strong positive staining of p-FLNC, its kinase AKT1, and p-ERK1/2, a key protein in the ERK signaling cascade that regulates the growth and differentiation of cells; in contrast, the chemotherapy-sensitive specimens showed negative or weak positive staining of the above proteins (Additional file 5). Similar to the western blot analysis results, the level of p-JNK was higher in the chemotherapy-sensitive specimens, and no significant difference was found with respect to p-P38. Altogether, the above results suggest that activated MAPK signaling in the process of rhIL-6-induced chemoresistance mainly occurs through p-ERK1/2 and p-JNK, but not p-P38. ERK1/2 inhibitors and the activation of JNK can be considered strategies to overcome chemoresistance to lobaplatin in osteosarcoma.

In summary, in this study, the global phosphorylation patterns of SaOS-2 osteosarcoma cells treated with rhIL-6 prior to lobaplatin were characterized. Based on a previous study that revealed reduced sensitivity in osteosarcoma cells after rhIL-6 treatment, we further found that p-FLNC can function as a lobaplatin resistance-related target in osteosarcoma cells. However, because a phosphoantibody was not available for all upregulated phosphoproteins, we did not detect additional phosphorylated proteins. We aimed to further verify other phosphoproteins after ensuring the reliability of the corresponding antibodies in order to reveal more possibilities related to drug resistance induced by IL-6 in osteosarcoma. Additionally, larger patient cohorts will be used to further evaluate this phosphoprotein and its kinase in lobaplatin-treated osteosarcoma specimens.

## Conclusion

In this study, we conducted a comprehensive analysis of the phosphoprotein profiles of chemoresistant and chemosensitive osteosarcoma cells induced by rhIL-6. p-FLNC, its kinase AKT1, and MAPK signaling were identified to play pivotal roles in this process. Further western blot analysis and IHC verification proved that JNK and ERK signaling, which were activated in rhIL-6-induced chemoresistant osteosarcoma cells, were the main contributors, while p38 signaling was not involved. This study provides an experimental basis for chemoresistance to lobaplatin at the post-translational level and reveals potential molecular targets for increasing chemosensitivity in osteosarcoma.

## Supplementary Information

**Additional file 1**. Hierarchical clustering of the differentially expressed phosphoproteins in SaOS-2 osteosarcoma cells between the Lob group and the Con group. Each group contained three biological replicates. In total, 1,815 phosphoproteins with significantly differential expression (specifically, 874 upregulated and 941 downregulated phosphoproteins) were identified (fold change > 1.2, *p* < 0.05).

**Additional file 2**. Clinical characteristics of osteosarcoma patients treated with platinum-based chemotherapy.

**Additional file 3**. Clinical characteristics of osteosarcoma patients and the correlation with chemotherapy sensitivity.

**Additional file 4**. Quantification of the expression of FLNC, ERK1/2, JNK, and P38 by immunohistochemistry staining.

**Additional file 5**. Kaplan-Meier overall survival curves comparing osteosarcoma patients with high and low p-FLNC expression levels (*n* = 40, *p* < 0.05)

## Data Availability

The MS proteomics data were deposited in the ProteomeXchange Consortium via the PRIDE [40] partner repository with the following ProteomeXchange dataset identifier: PXD025970.

## Abbreviations

rhIL-6: Recombinant human IL-6
GO: Gene Ontology
KEGG: Kyoto Encyclopedia of Genes and Genomes
DTT: Dithiothreitol
UA: Uric acid
HCD: Higher energy collisional dissociation
AGC: Automatic gain control
IT: Injection time
KO: KEGG Orthology
KOALA: KEGG Orthology and Links Annotation
SDS-PAGE: Sodium dodecyl sulfate–polyacrylamide gel electrophoresis
MS: Mass spectrometry
DEPs: Differentially expressed phosphoproteins
TMT: Tandem mass tag
PPI: Protein-protein interaction
p-ERK1/2: P-extracellular signal-regulated kinase 1/2
IHC: Immunohistochemical
HE: Hematoxylin and eosin
TLR: Toll-like receptor
NLR: NOD-like receptor
