# Supplementary material for: Quantitative phosphoproteomic analysis reveals chemoresistance-related proteins and signaling pathways induced by rhIL-6 in human osteosarcoma cells
Source: Cancer Cell Int. 2021 Oct 30;21:581. doi: 10.1186/s12935-021-02286-z (PMC8557500; doi:10.1186/s12935-021-02286-z)

**Additional Files**

**Additional file 1. Hierarchical clustering of the differentially expressed phosphoproteins in SaOS-2 osteosarcoma cells between the Lob group and the Con group. Each group contained three biological replicates. In total, 1,815 phosphoproteins with significantly differential expression (specifically, 874 upregulated and 941 downregulated phosphoproteins) were identified (fold change>1.2, p<0.05).**


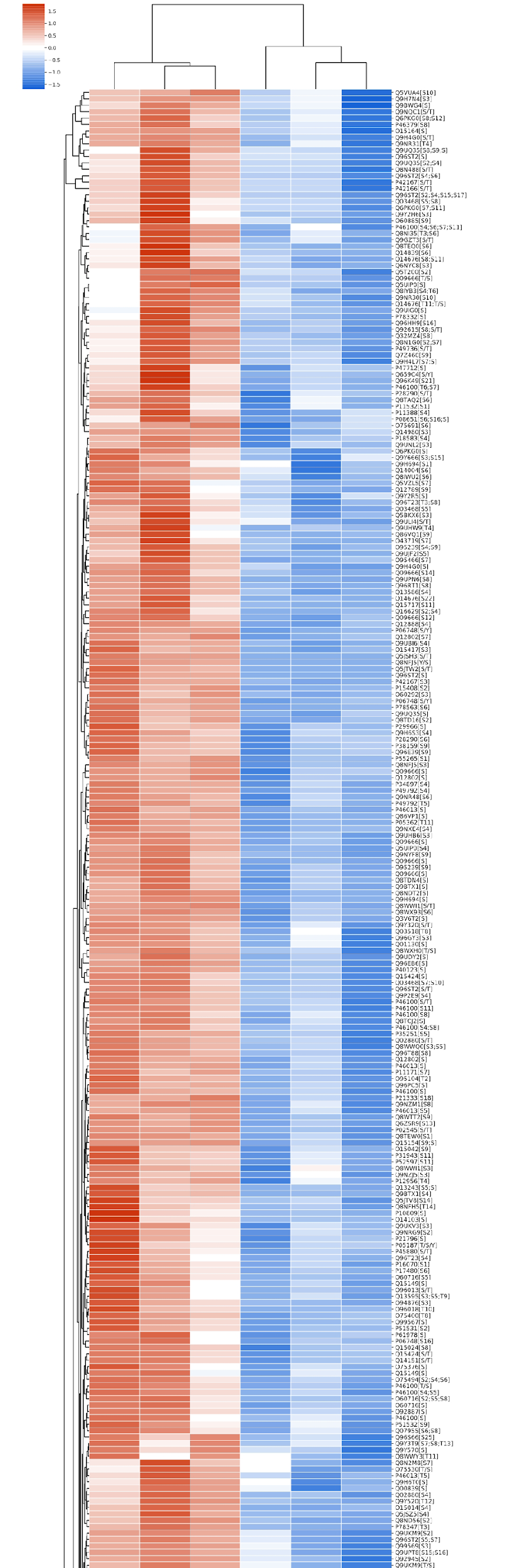


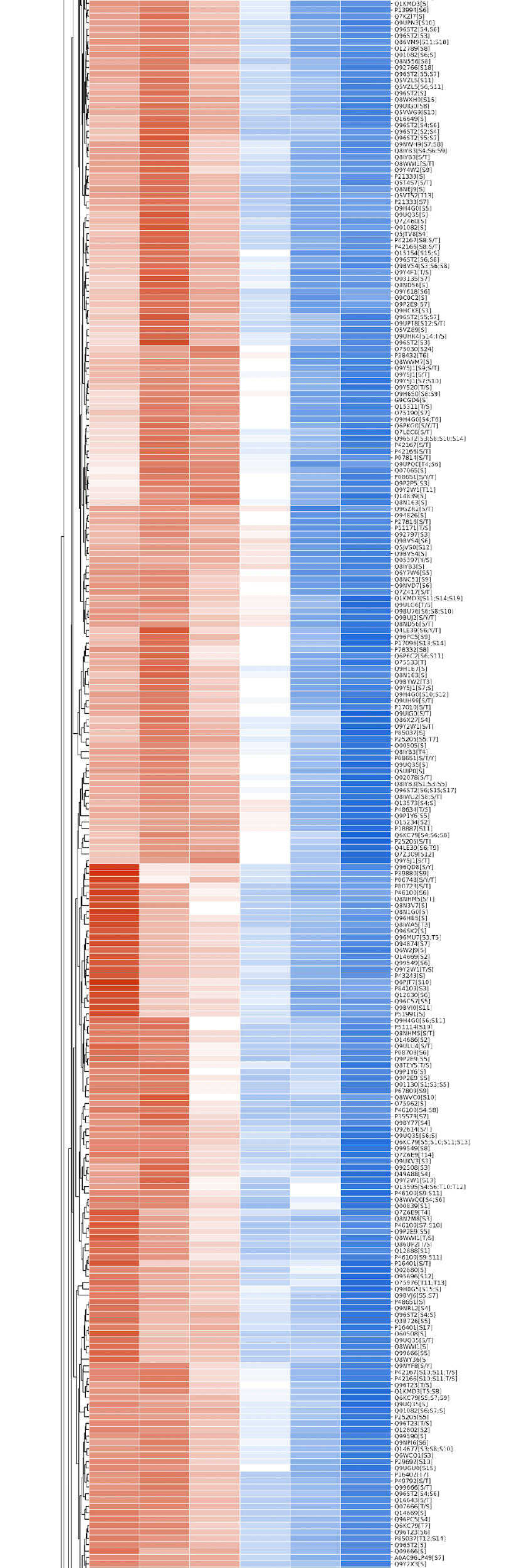


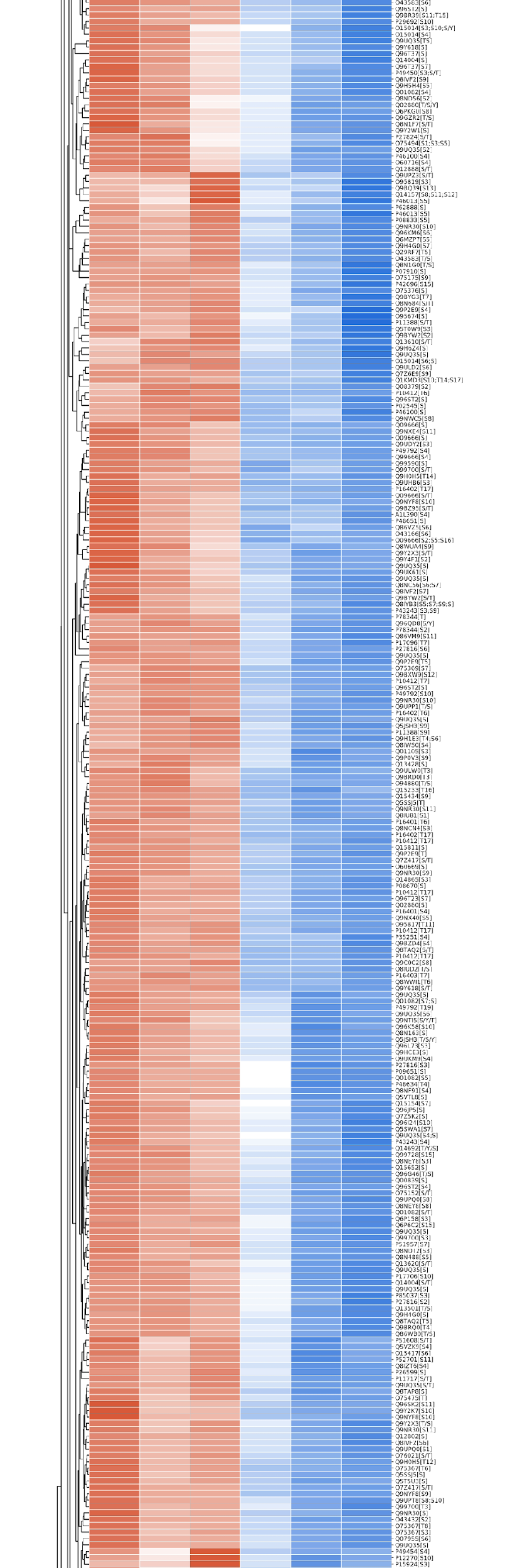


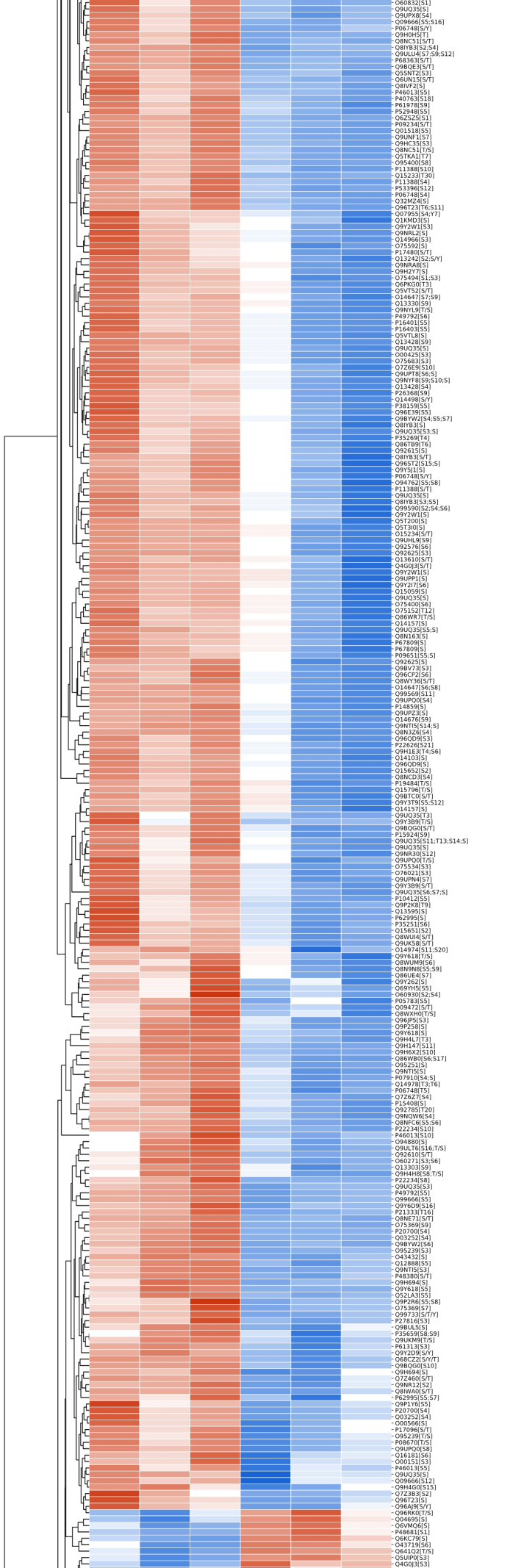


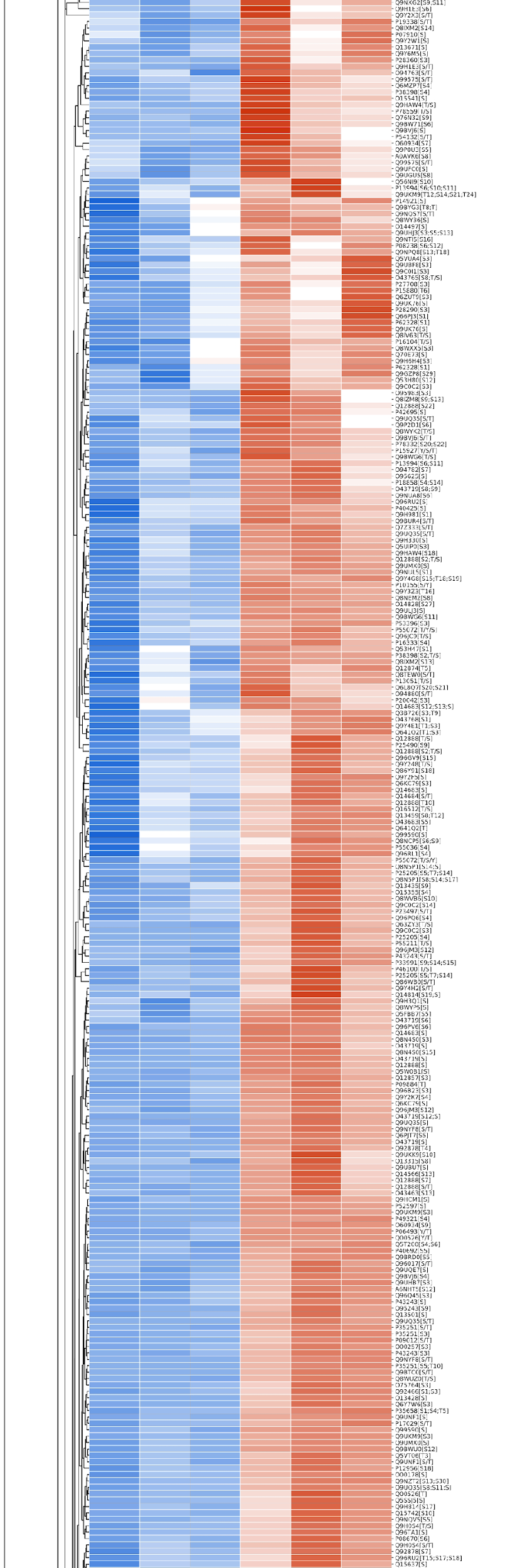


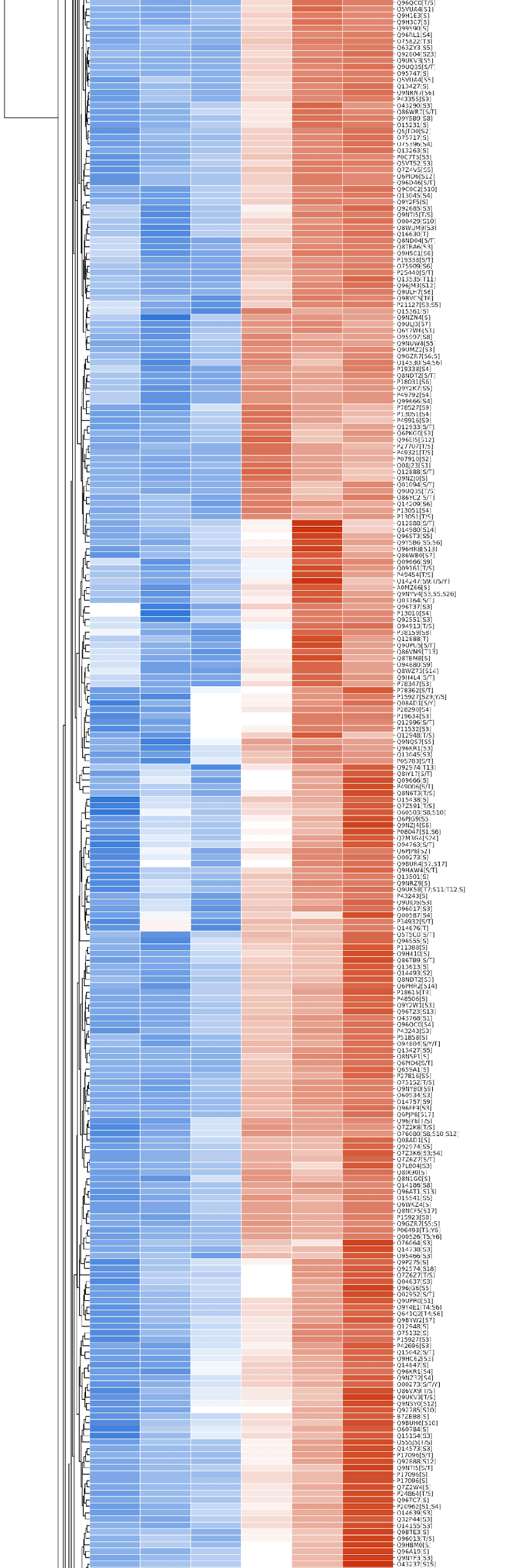


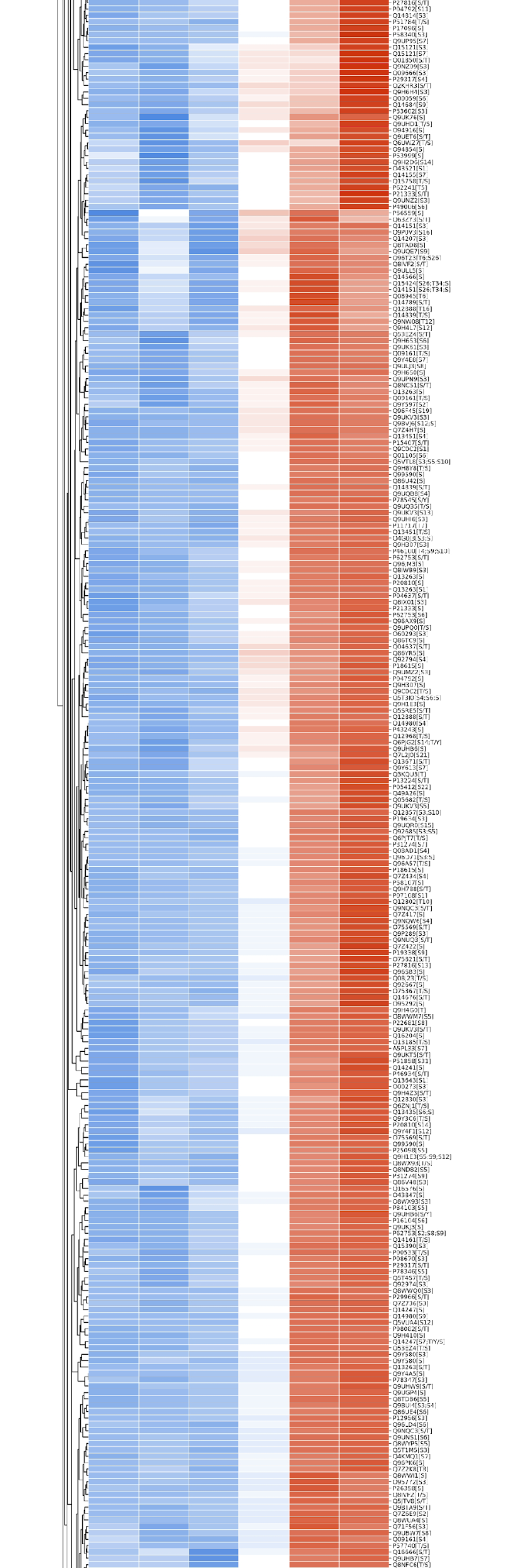


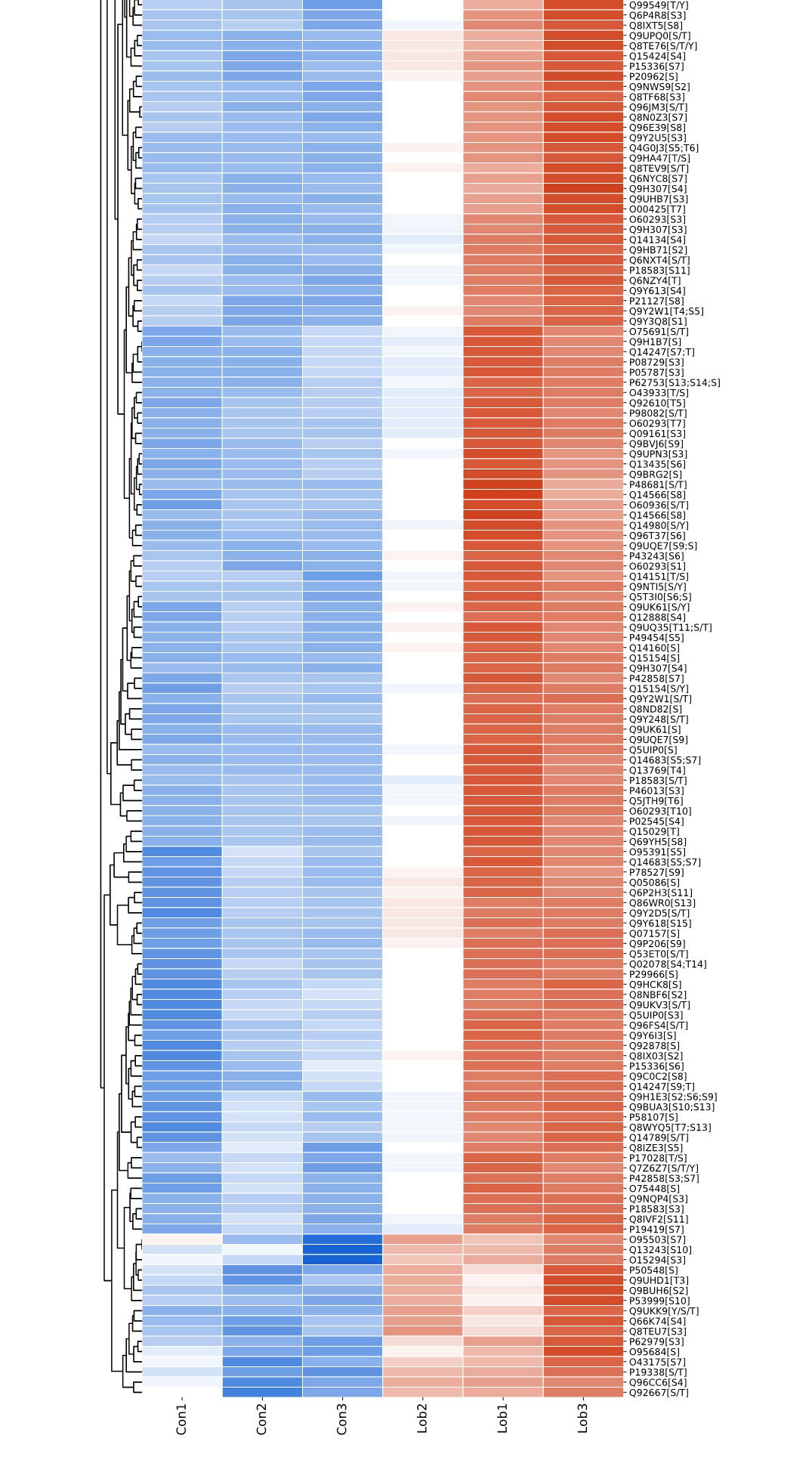

Supplement: Supplementary file 1 — Additional file 1. Hierarchical clustering of the differentially expressed phosphoproteins in SaOS-2 osteosarcoma cells between the Lob group and the Con group. Each group contained three biological replicates. In total, 1,815 phosphoproteins with significantly differential expression (specifically, 874 upregulated and 941 downregulated phosphoproteins) were identified (fold change > 1.2, p < 0.05). [file 12935_2021_2286_MOESM1_ESM.docx]
