# Supplementary figures and images for: Quantitative phosphoproteomic analysis reveals chemoresistance-related proteins and signaling pathways induced by rhIL-6 in human osteosarcoma cells
Source: Cancer Cell Int. 2021 Oct 30;21:581. doi: 10.1186/s12935-021-02286-z (PMC8557500; doi:10.1186/s12935-021-02286-z)

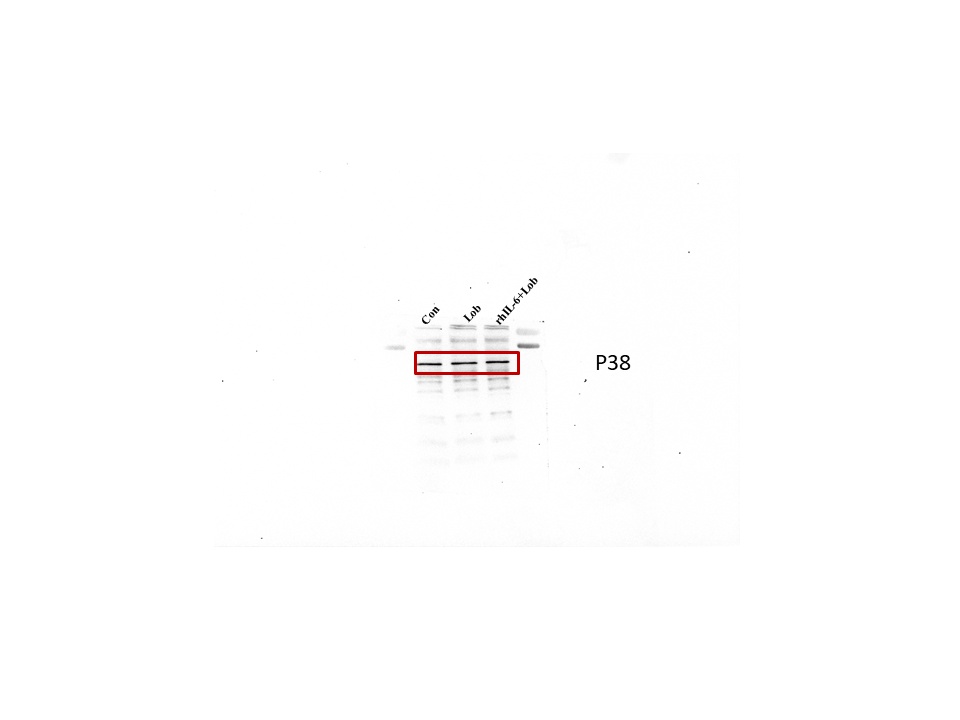

Supplement: Supplementary file 2 — Additional file 2. Clinical characteristics of osteosarcoma patients treated with platinum-based chemotherapy. [file 12935_2021_2286_MOESM2_ESM.tif]

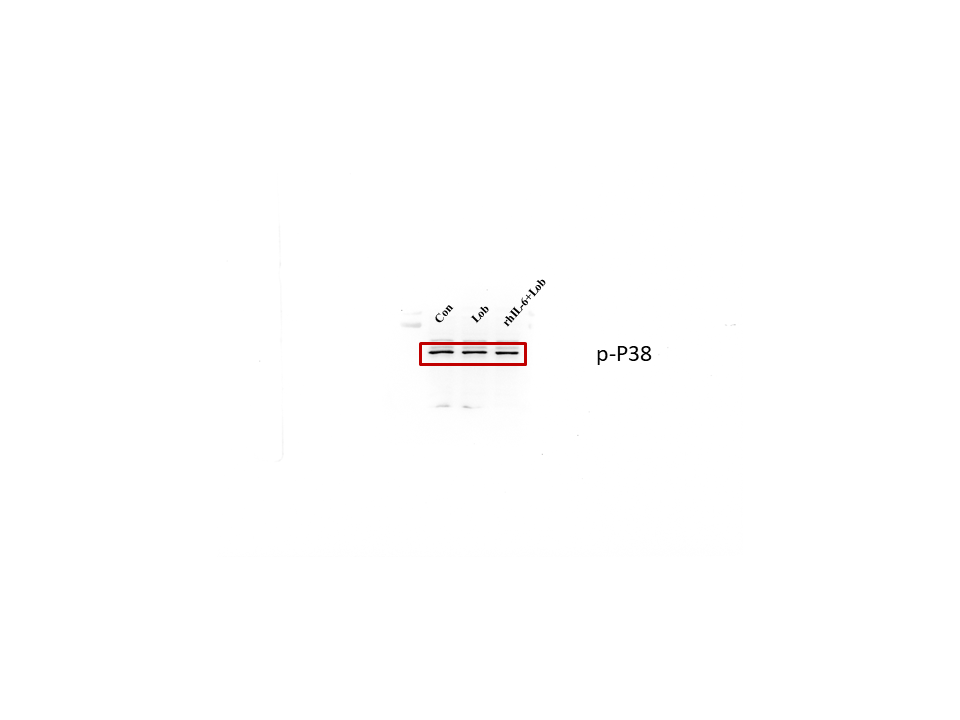

Supplement: Supplementary file 3 — Additional file 3. Clinical characteristics of osteosarcoma patients and the correlation with chemotherapy sensitivity. [file 12935_2021_2286_MOESM3_ESM.tif]

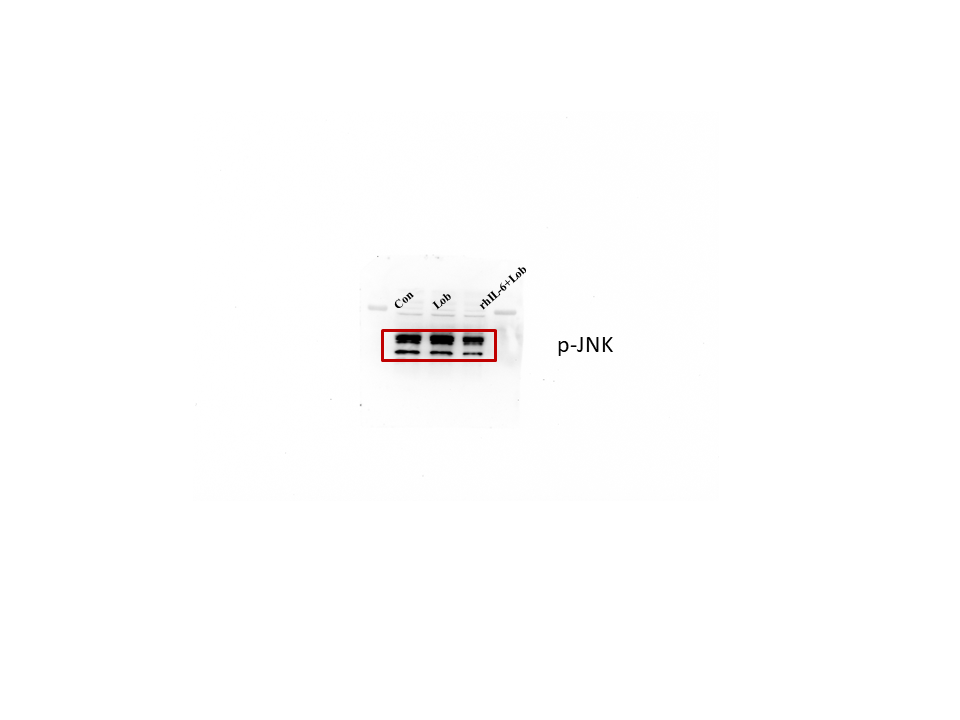

Supplement: Supplementary file 4 — Additional file 4. Quantification of the expression of FLNC, ERK1/2, JNK, and P38 by immunohistochemistry staining. [file 12935_2021_2286_MOESM4_ESM.tif]

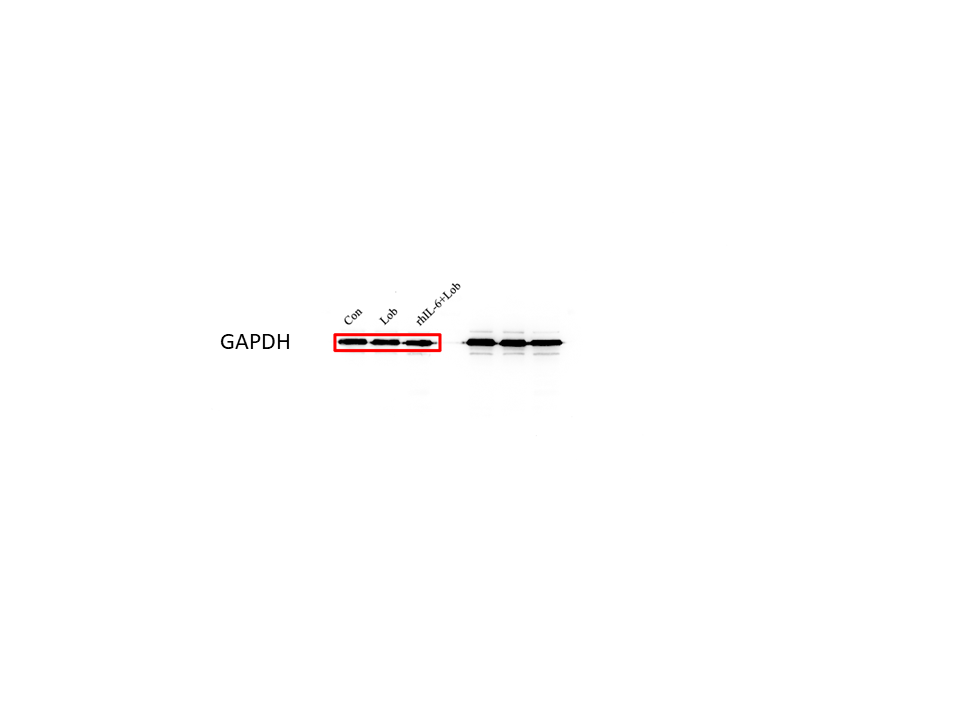

Supplement: Supplementary file 5 — Additional file 5. Kaplan-Meier overall survival curves comparing osteosarcoma patients with high and low p-FLNC expression levels (n = 40, p < 0.05) [file 12935_2021_2286_MOESM5_ESM.tif]
